# Supplementary material for: Piezo-Sensitive Fabrics from Carbon Black Containing Conductive Cellulose Fibres for Flexible Pressure Sensors
Source: Materials (Basel). 2020 Nov 16;13(22):5150. doi: 10.3390/ma13225150 (PMC7696665; doi:10.3390/ma13225150)
Supplement: Supplementary file 1 [file materials-13-05150-s001.pdf]

materials

MDPI

Supplementary Information

Piezo-Sensitive Fabrics from Carbon Black Containing Conductive Cellulose Fibres for Flexible Pressure Sensors

Julia Ullrich [1], Martin Eisenreich [1], Yvonne Zimmermann [1], Dominik Mayer [2], Nina Koehne [2], Jacqueline F. Tschannett [3], Amalid Mahmud-Ali [3] and Thomas Bechtold [3,*]

[1] Textilforschungsinstitut Thüringen-Vogtland e.V., Zeulenrodaer Straße 42, D-07973 Greiz, Germany; j.ullrich@titv-greiz.de (J.U.); m.eisenreich@titv-greiz.de (M.E.); y.zimmermann@titv-greiz.de (Y.Z.)
[2] Kelheim Fibres GmbH, Regensburger Straße 109, D-93309 Kelheim, Germany; Dominik.Mayer@kelheim-fibres.com (D.M.); Nina.Koehne@kelheim-fibres.com (N.K.)
[3] Research Institute of Textile Chemistry and Textile Physics [†], Leopold-Franzens-University of Innsbruck, Hoechsterstraße 73 A-6850 Dornbirn, Austria; Jacqueline.Tschannett@uibk.ac.at (J.F.T.); Amalid.Mahmud-Ali@uibk.ac.at (A.M.-A)
* Correspondence: thomas.bechtold@uibk.ac.at; Tel: +43 (0) 5572-28533
[†] Member of EPNOE–European Polysaccharide Network of Excellence, www.epnoe.eu

Received: 18 October 2020; Accepted: 13 November 2020; Published: date

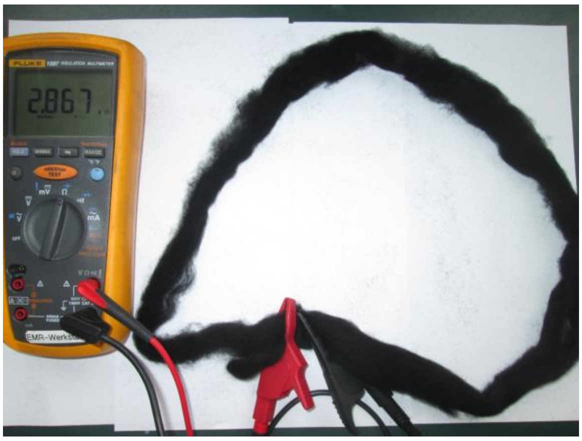

Figure S1. Rotor ring and measurement of resistance measurement with multi-meter.

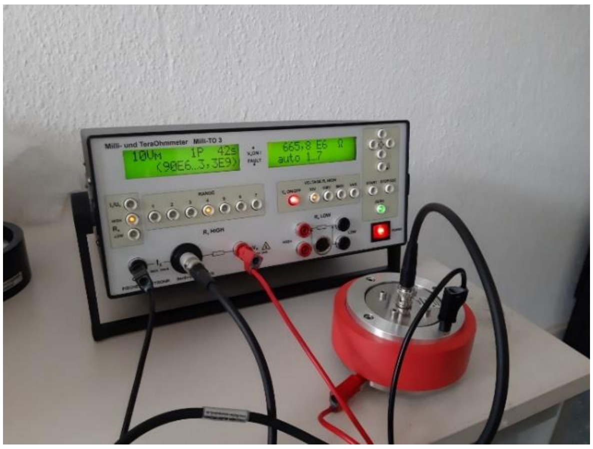

Figure S2. Experimental setup for the measurement of volume resistance.

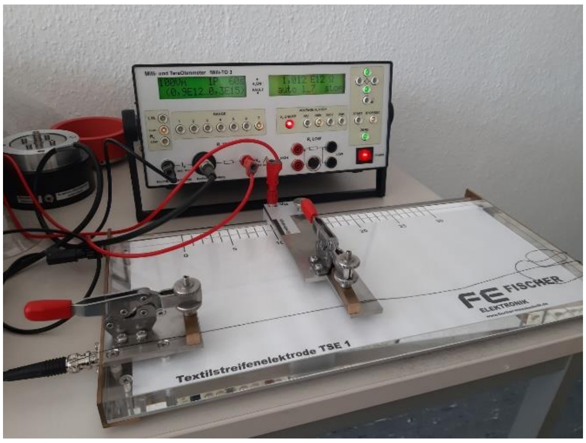

Figure S3. Experimental setup for the measurement of yarn resistance.

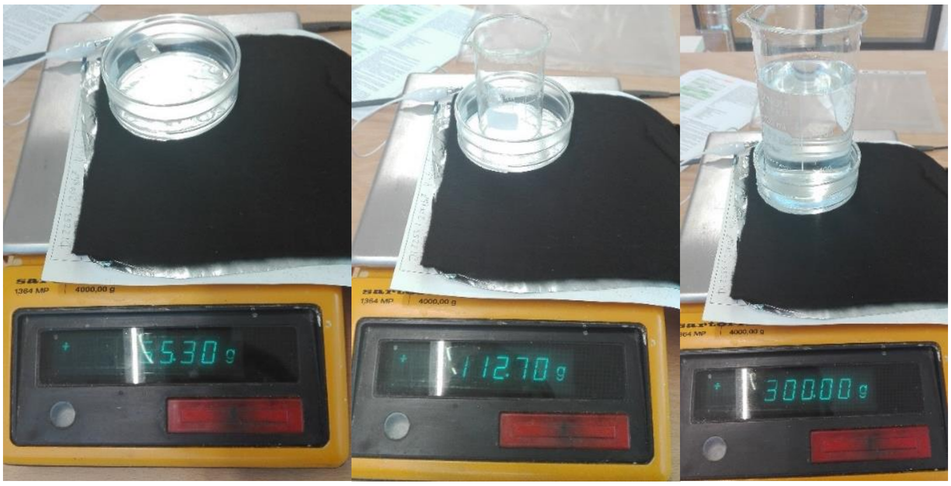

Figure S4. Experimental setup for the measurement of resistance as function of pressure.

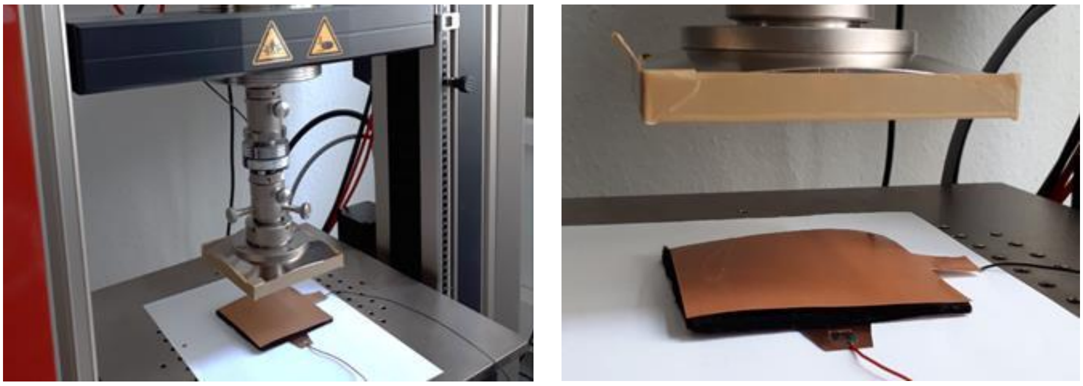

Figure S5. Experimental setup for the cyclic load/release experiments in a tensile testing unit.

Publisher's Note: MDPI stays neutral with regard to jurisdictional claims in published maps and institutional affiliations.

© 2020 by the authors. Licensee MDPI, Basel, Switzerland. This article is an open access article distributed under the terms and conditions of the Creative Commons Attribution (CC BY) license (http://creativecommons.org/licenses/by/4.0/).
